# Treating Hematologic Malignancies During a Pandemic: Utilizing Telehealth and Digital Technology to Optimize Care

**DOI:** 10.3389/fonc.2020.01183

**Published:** 2020-06-26

**Authors:** Adam F. Binder, Nathan R. Handley, Lindsay Wilde, Neil Palmisiano, Ana Maria Lopez

**Affiliations:** Department of Oncology, Sidney Kimmel Cancer Center, Thomas Jefferson University Hospital, Philadelphia, PA, United States

**Keywords:** telehealth, digital technology, care at home, patient centered care, healthcare innovation

## Abstract

In late January 2020, Severe Acute Respiratory Syndrome Coronavirus-2 (SARS CoV-2) was reported as an outbreak in Wuhan, China. Within 2 months it became a global pandemic. Patients with cancer are at highest risk for both contracting and suffering complications of its resultant disease, Coronavirus 19 (COVID-19). Healthcare systems across the world had to adapt quickly to mitigate this risk, while continuing to provide potentially lifesaving treatment to patients. Bringing care to the home through the use of telehealth, home based chemotherapy, and remote patient monitoring technologies can help minimize risk to the patient and healthcare workers without sacrificing quality of care delivered. These care models provide the right treatment, to the right patient, at the right time, *in the right place*. Whether these patient-centered models of care will continue to be embraced by key stakeholders after the pandemic remains uncertain.

## Introduction

In late January 2020, the outbreak of Severe Acute Respiratory Syndrome Coronavirus-2 (SARS CoV-2) and its resultant disease, Coronavirus 19 (COVID-19), was reported as an emerging epidemic in Wuhan, China. Within 2 months, COVID-19 became a global pandemic (1). Individuals at highest risk for severe complications tend to be older (>65 years old) with multiple comorbidities (2). Patients with hematologic malignancies fall into this category; these patients have a median age of diagnosis of 70.6 years (3) and many are immunosuppressed due to either the cancer itself or to the treatment regimen they are receiving. Patients with hematologic malignancies are at even high risk for infection as a result of immune dysregulation that can persist even years after treatment is completed (4). In addition, patients with hematologic malignancies have frequent contact with the health care system, increasing their risk for exposure and infection (5). As a result of increased susceptibility and contact with the healthcare system, patients with hematologic malignancies appear to have a higher incidence of COVID-19 infection and severe complications (6–8).

In order to minimize risk to the population at large, governments have implemented mitigation policies that include mandatory shelter in place orders and social distancing rules. These measures have challenged cancer centers to develop strategies to continue to provide appropriate care while minimizing risk of infection for both patients and healthcare teams. These strategies include workflow processes to create an environment to allow for social distancing; operational models to ensure access to appropriate screening, testing, and personal protective equipment; treatment modifications, such as personalizing timing of cancer treatment based on emerging national and international guidelines and the specific characteristics of the patient's disease, underlying morbidities, and risk; and care decentralization, enabling patients to shelter in place while continuing to receive the care they need (9).

Decentralized care—bringing care to the patient, rather than the traditional approach of bringing the patient to care—takes advantage of the growing capabilities of health systems and their patients to utilize telecommunications technology to enable health care delivery *to* the patient. Tools for enabling decentralized care include (10) telehealth visits, home based care, and remote patient monitoring. Herein, we will examine the benefits, barriers and considerations for each of these components of decentralized care with a focus on how they are important in providing appropriate care during the current pandemic and how they can become integrated into standard of care in the future.

### Telehealth Visits

Telehealth visits (clinician-patient visits using two-way, real-time audiovisual or audio-only technology, or physician-physician synchronous or asynchronous consultations) can minimize healthcare exposure. These visits can be utilized for new or routine follow-up of patients with hematologic malignancies (11). Telehealth visits not only protect patients from exposure risk, but also mitigate exposure for the healthcare teams. According to a CDC report, as of April 15th, 2020, of the healthcare professionals who have contracted COVID-19 in the United States, about 55% of those cases were thought to be due to work-related exposures (12). Ensuring that providers are only seeing patients when it will truly change management decisions may reduce transmission of SARS-CoV-2.

### Outpatient

While telehealth visits have been utilized for decades prior to the pandemic (13, 14), their use has dramatically increased following the onset of COVID-19 (10). At Sidney Kimmel Cancer Center at Thomas Jefferson University, the use of telehealth increased from <2.0 to over 50% of all hematologic malignancy visits within weeks. This rapid scale was possible as a result of an existing robust telehealth program within the Jefferson Health enterprise. In audiovisual visits, one can perform almost all aspects of an in-person visit. Physical exams are dynamic. If the patient is alone, exam may be limited to observational only. If the patient has a caregiver with them, one can observe patient response to certain manipulations of joints or palpation of specific areas. With the right digital tools, such as remote patient monitoring devices to obtain vital signs as well as a digital stethoscope to auscultate the heart and lungs, along with a caregiver to perform certain palpation techniques, one can complete a thorough physical exam (15). These visits can be used for routine follow-up (16) or acute care (17) with an overall positive patient experience. Unpublished post-visit survey data (completed within 2 weeks of the telehealth visit) from our institution revealed that for all patients with cancer, 99% of patients were satisfied with their telehealth visit; 91% of patients agreed our telehealth visit system (JeffConnect) was easy to use; 94% said they would use telehealth visits again; 87% felt it provided the same care as an in person visit; on a 10-point scale, average likelihood to recommend JeffConnect to a friend or colleague was 9.16 (net promoter score 68, *n* = 97).

To further promote the use of telehealth visits, the Center for Medicare and Medicaid Services (CMS) has liberalized reimbursement regulations for telehealth services during the pandemic (18). Previously, CMS reimbursements for telehealth visits were restricted to those over the age of 65 and who lived in rural communities. In addition, these visits had to take place in a rural health care facility and only within an established patient-physician relationship. Now, telehealth visits can occur from any location for either new or follow-up clinic visits. Both audio-visual and audio-only telehealth visits are now reimbursed on par with a face-to-face visit. Transitioning patients to telehealth visits, particularly those on oral chemotherapy or post-treatment follow up, can reduce an individual patient's risk for exposure, while at the same time minimizing exposure risk for the clinician and preserving personal protective equipment (PPE) (9).

### Inpatient

Inpatient teleconsultations were initiated at our institution via a telehealth HIPAA-compliant system that allows consulting teams to perform audiovisual telehealth visits in the hospital in order to minimize exposure to patients and staff. While this model is well-established for tele-psychiatry (19), intensive care (20), and pediatrics (21) it is increasingly used in cancer care (15). Patients with COVID-19 can continue to receive subspecialty care without healthcare workers exposing themselves unnecessarily. In addition, at a time when patients' families are not allowed in the hospital, and patients in the hospital feel increasingly isolated, it provides an opportunity for family members and caregivers to simultaneously connect with healthcare workers and the patient in order to continue to be involved in healthcare decision making.

### Physician-Physician

Physician to physician directed synchronous and asynchronous models enable information exchange between clinicians without physical contact (22–26). It also often reduces the number of patient-physician encounters. In the outpatient setting at the VA, use of asynchronous hematology e-consultative services reduced the number of face-to-face referrals (27). In hematologic malignancies, asynchronous e-consultation may be particularly useful in reducing referrals and patient evaluations for diseases, such as early stage CLL, asymptomatic indolent lymphomas, myeloproliferative neoplasms, or plasma cell dyscrasias, where the initial work up can be completed by the referring physician and active surveillance is often the recommended standard of care approach.

For patients with newly diagnosed hematologic malignancies, timely evaluation and treatment is central to optimizing outcomes. With new CMS regulations allowing for initial evaluation via telehealth visits, patients can complete their initial visit via telehealth, while in-person visits can be reserved for those who are acutely symptomatic or in need of diagnostic interventions such as a bone marrow biopsy or lumbar puncture. For those who establish care via a telehealth visit and need to initiate treatment, tele-consenting models may facilitate starting therapy (28). Initial data suggest that patients are satisfied with tele-consenting (29).

Implementation of these programs is not without challenges. Lack of reliable internet access, lack of education on how to use smart devices, and lack of a smart device are major barriers to widespread implementation of telehealth. A previous study revealed that those, who are older, are of minority race or ethnicity or with less than a college education are less likely to have access to home internet (30). With the right support, however, these limitations may be overcome. At Thomas Jefferson University, in response to COVID-19, a telehealth task force was implemented. This group has worked diligently to ensure every patient has the capabilities to complete a video visit. This support ranges from taking patients through a step by step process for setting up the patient portal to providing a free tablet computer to complete the necessary visits. While these efforts require significant resources, they have been coupled by a rise in the percentage of patients who have been able to successfully complete a video visit.

While telehealth strategies are an effective way of establishing care and initiating treatment during the current pandemic, they may prove to be important components of standard of care in the future. Additional research into the impact of telemedicine on the patient-physician relationship, time to treatment, cost of care, patient and clinician satisfaction, and outcomes is needed.

## Home Based Therapy

Home based therapy is the treatment of patients in the home setting either with anti-neoplastic agents or supportive therapies. For some patients with hematologic malignancies, such as acute leukemia or high-grade B cell lymphomas, treatment cannot be safely postponed and carries significant nosocomial infection risks by way of exposure to carriers (many of whom may be asymptomatic) (31). Home based chemotherapy can mitigate this risk.

While not well-established in the US, models for the delivery of home chemotherapy exist globally and have been shown to be safe, patient centered, and more cost effective when compared to the hospital or infusion center (32–35). However, home chemotherapy comes with unique challenges. For instance, to care for the same number of patients, significant staffing changes must occur, as nurses must travel from one home to the next as opposed to simultaneously caring for patients in adjacent infusion chairs. Other logistical challenges exist. USP-800 regulations must be followed for compounding, storing, administering and disposing of chemotherapeutic agents (36) and drug stability must be considered so medications are delivered and administered in the appropriate time frame. Tracking of home chemotherapy administration in the electronic medical record must also be completed accurately, reliably, and in a timely fashion. However, creative solutions for these problems can be developed. For example, for some agents, such as injectable medications (i.e., bortezomib, rituximab-hycela), a telehealth infusion model could exist in which a chemotherapy certified nurse observes self-administration and proper disposal via a telehealth visit.

In addition to chemotherapy administration, home blood product transfusion can also be implemented to minimize exposure to the healthcare system. Some patients with myelodysplastic syndrome or acute leukemia require frequent, often biweekly, transfusions and are concurrently neutropenic, greatly increasing their risk for infection. Home-based blood transfusion programs are uncommon in the US but are more widely implemented in Europe and are safe, effective, and preferred by patients (37–39). If more broadly implemented, patients with hematologic malignancies would benefit greatly from ongoing programs that are able to provide these therapies at home. The current COVID-19 pandemic may be the catalyst for change that has been needed to allow for these services to become standard of care.

### Remote Patient Monitoring

Remote patient monitoring (RPM) is the use of web-based systems and wearable sensors to monitor health data. Utilization of these digital technologies during the pandemic can allow clinical teams to closely monitor patients with hematologic malignancies.

For patients with advanced cancer who are receiving chemotherapy, monitoring symptoms via electronic patient reported outcomes (ePROs) has been shown to improve symptom management, decrease acute care utilization, and improve overall survival (40–42). Reducing acute care utilization is particularly important during the COVID-19 pandemic as acute care resources may become scarce and resource allocation may become challenging. Ongoing studies are evaluating the applicability of ePROs in hematologic malignancies (43). While initial results are promising, implementation can be challenging and requires expertise (44). Digital health literacy varies based on age and education level (30) and understanding a practice's patient population is important when considering the various models for ePRO implementation.

In addition to monitoring patient's reported symptoms, continuous biometric data monitoring in combination with standard interventions has shown some promise in supplementing home based care, though results have been mixed to date (45–49). Most studies have evaluated integration of activity trackers or continuous temperature monitoring into patient care. Activity trackers have been studied to assess effects of treatment; baseline activity prior to treatment has been shown to be prognostic (48–50). With respect to temperature monitoring, a pilot study demonstrated that inpatient continuous temperature monitoring in neutropenic patients identified fever 11.4 h earlier than routine intermittent temperature monitoring (51). Multiple RPM studies are ongoing^1^^,^^2^. While there is significant promise in the field of RPM, many questions regarding its use remain, and how it will apply to patient with hematologic malignancies is unclear. The COVID-19 pandemic may highlight the potential utility of RPM and accelerate research in this area.

## Conclusion

The COVID-19 pandemic has rapidly changed care delivery. The swift adoption of decentralization strategies like telehealth visits, home-based care, and remote patient monitoring can allow physicians treating hematologic malignancies to maintain care while mitigating the risk of nosocomial SARS-COV-2 infection. These tools can be utilized across the spectrum of care from, prevention (26) to palliation (52), to reduce the care burden as well as decrease exposure risk for patients and health care teams. Decentralization of care can provide a patient-centered approach that follows a precision medicine algorithm with an extra feature: providing the right treatment, to the right patient, at the right time and *in the right place*. Whether these strategies will continue to be wholeheartedly embraced by key stakeholders—health care professionals, patients, and payers—as the pandemic subsides remains to be seen.

## Data Availability Statement

The original contributions presented in the study are included in the article/supplementary material, further inquiries can be directed to the corresponding author/s.

## Author Contributions

All authors contributed to the writing and editing of this manuscript.

## Conflict of Interest

The authors declare that the research was conducted in the absence of any commercial or financial relationships that could be construed as a potential conflict of interest.

## Footnotes

^1^ClinicalTrials.gov. Available online at: https://clinicaltrials.gov/ct2/results?cond=remote+patient+monitoring&term=oncology&cntry=&state=&city=&dist= (accessed June 1, 2020).

^2^ClinicalTrials.gov. *Remote Outpatient Temperature Monitoring for Early Detection of Febrile Neutropenia After Chemotherapy (REMEDY)*. Available online at: https://clinicaltrials.gov/ct2/show/NCT04081753 (accessed June 1, 2020).
